# Low seasonal variation in greater mouse-eared bat (*Myotis myotis*) blood parameters

**DOI:** 10.1371/journal.pone.0234784

**Published:** 2020-07-07

**Authors:** Hana Bandouchova, Jan Zukal, Petr Linhart, Hana Berkova, Jiri Brichta, Veronika Kovacova, Aneta Kubickova, Ehdaa E. E. Abdelsalam, Tomáš Bartonička, Renata Zajíčková, Jiri Pikula

**Affiliations:** 1 Department of Ecology and Diseases of Zoo Animals, Game, Fish and Bees, University of Veterinary and Pharmaceutical Sciences Brno, Brno, Czech Republic; 2 Institute of Vertebrate Biology, Czech Academy of Sciences, Brno, Czech Republic; 3 Department of Botany and Zoology, Masaryk University, Brno, Czech Republic; 4 Institute of Biostatistics and Analyses, Masaryk University, Brno, Czech Republic; University of Minnesota, UNITED STATES

## Abstract

The greater mouse-eared bat (*Myotis myotis*) is a flagship species for the protection of hibernation and summer maternity roosts in the Western Palearctic region. A range of pathogenic agents is known to put pressure on populations, including the white-nose syndrome fungus, for which the species shows the highest prevalence and infection intensity of all European bat species. Here, we perform analysis of blood parameters characteristic for the species during its natural annual life cycle in order to establish reference values. Despite sexual dimorphism and some univariate differences, the overall multivariate pattern suggests low seasonal variation with homeostatic mechanisms effectively regulating haematology and blood biochemistry ranges. Overall, the species displayed a high haematocrit and haemoglobin content and high concentration of urea, while blood glucose levels in swarming and hibernating bats ranged from hypo- to normoglycaemic. Unlike blood pH, concentrations of electrolytes were wide ranging. To conclude, baseline data for blood physiology are a useful tool for providing suitable medical care in rescue centres, for studying population health in bats adapting to environmental change, and for understanding bat responses to stressors of conservation and/or zoonotic importance.

## Introduction

The greater mouse-eared bat’s (*Myotis myotis*) range of distribution covers most of the Western Palearctic region. Given its wide geographic range and abundance (present populations having recovered and stabilised from past declines), the species is now classified as of ‘Least Concern’ in the International Union for the Conservation of Nature’s Red List of Threatened Species [1]. On the other hand, it remains listed as a protected species in the Agreement on the Conservation of Populations of European Bats (UNEP/EUROBATS). Switching roosts during the annual cycle, it forms summer nursery colonies in either loft spaces of buildings or caves and utilises underground hibernacula in winter. The species is under threat throughout the year from damage to roost sites, habitat deterioration and use of insecticides [1].

Like other insectivorous bat species, the greater mouse-eared bat has adapted to northern temperate zone seasons by alternating physiological states associated with heterothermy and homeothermy. As a result, the bats are able to quickly change their body temperature, with temperatures during active flight reaching more than 40°C [2] and dropping to 0–12°C during hibernation torpor. During hibernation, the bats periodically re-warm for several hours and achieve normothermy for homeothermic mammals [3–5], though such full arousals may be aborted at any time before the body temperature reaches normothermy [6,7]. Such extreme fluctuations in body temperature are reflected in many of the bat’s physiological features, including blood characteristics [8–10].

Many of the physiological aspects and conditions associated with the bat’s seasonal life cycle make great demands on the maintenance of blood parameters [11,12]. For example, bats may ingest large amounts of food when foraging, but fast for long periods when food is unavailable, resulting in considerable changes in body mass. Further, the bat’s water and electrolyte balance may be challenged by rapid digestion and nutrient absorption following large meals, as well as through evaporative water loss through the respiratory tract and the large naked flight membranes. Reduction and activation of the bat’s metabolism requires nutrient storage or mobilisation, respectively, while switching between protein, carbohydrate and lipid metabolisms will also cause changes in blood chemistry. Finally, gestation and lactation supporting the pre- and post-natal development and growth of offspring imposes severe demands on the female [13].

Pathological conditions, infections and subclinical diseases may also induce responses manifested in the blood profile [14–16]; and this must be taken into account when establishing reference ranges typical for healthy animals. Of all the European bat species, the greater mouse-eared bat shows the highest prevalence and infection intensity of the white-nose syndrome fungus, *Pseudogymnoascus destructans* [17–21]. While tolerance to *P*. *destructans* infection has evolved in the Palearctic region, allowing survival under pathogen pressure [21,22], hibernating greater mouse-eared bats show homeostatic disruption manifested as mild metabolic acidosis, decreased glucose and peripheral blood eosinophilia [15] when infection intensity exceeds 300 skin lesions on both wings.

While blood analysis is a standard tool for examining general states of health and detecting and recognising pathological conditions through comparison with reference ranges, we lack comprehensive data for most bat species [23]. In order to establish reference blood profiles for the greater mouse-eared bat we collected samples immediately following bat capture and examined these in the field using on-site analysers, thereby obtaining representative data for physiological conditions in natural roosting habitats. We then compare this data with that collected during hibernation, lactation and swarming in order to test our prediction of high blood parameter variation caused by alterations in physiological states and the demanding extremes imposed by the seasonal life cycle. The results of this study provide useful information for research into wildlife diseases, with implications for conservation medicine.

## Material and methods

### Ethics statement

Collection of blood and the sampling of bats in the field was performed in accordance with Czech Law No. 114/1992 on Nature and Landscape Protection, based on permits 1662/MK/2012S/00775/MK/2012, 866/JS/2012 and 00356/KK/2008/AOPK issued by the Agency for Nature Conservation and Landscape Protection of the Czech Republic. The Ethical Committee of the Czech Academy of Sciences approved all experimental procedures (Project No. 169/2011). The authors were all authorised to handle free-ranging bats in agreement with Czech Certificate of Competency No. CZ01341 (§17, Act No. 246/1992). We made every effort to minimise the impact of disturbance, handling stress and sampling procedure duration. All bats were released at the capture site within an hour of capture.

### Bat sampling

**Fig 1:**
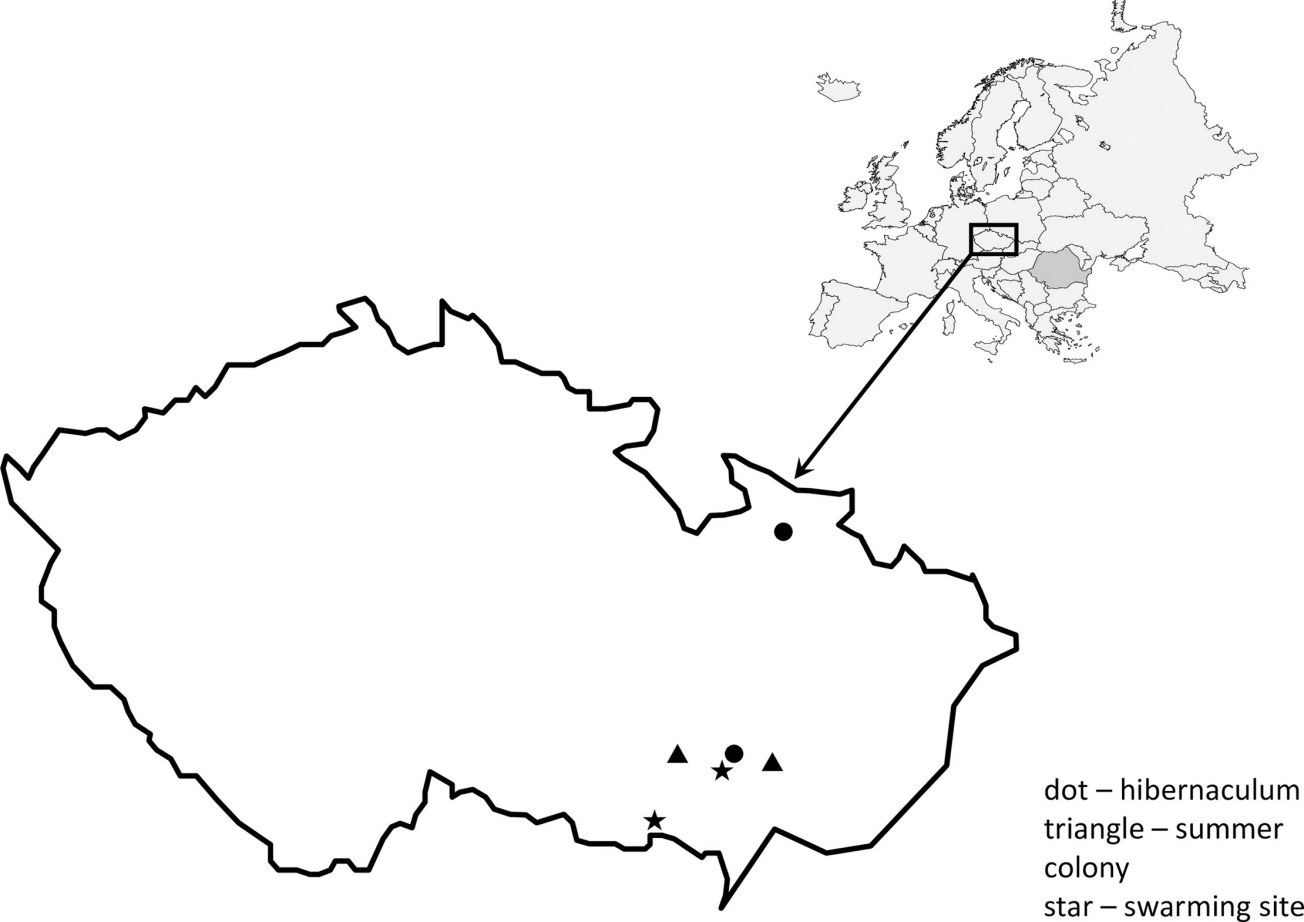
Map of localities sampled in the Czech Republic.

In 2015, a total of 98 bats were sampled at six localities in the Czech Republic (Fig 1), including two summer colonies (church attics in Doubravník [geographical coordinates 49.4256094N, 16.3518378E] and Otaslavice [49.3848658N, 17.0676067E]; sampling in July), two swarming sites (the Kateřinská cave [49.3607006N, 16.7102508E] and pseudo-karst caves [Ledové sluje] in the Podyji National Park [48.8846406N, 15.8448672E]; sampling in September and October) and two hibernacula (the Šimon and Juda mines [50.0487603N, 17.2966989E] and the Sloupsko-Šošůvské caves [49.4104556N, 16.7390147E]; sampling in April). Ambient temperatures ranged from 4.5 to 7.5°C at hibernacula, 35 to 55°C at church attics containing summer colonies and 12 to 20°C at swarming sites. While hibernating bats were in torpor with body temperatures close to the ambient temperature, swarming and lactating bats were active maintaining homeothermy. Bats were sampled as hibernating (winter), lactating (summer) and swarming (autumn). Spring in the temperate region of Central Europe is the season of bat movements from wintering sites to summer colonies, making blood collection from sufficient numbers of individuals impossible. The same difficulty concerns males staying solitary in spring and summer. The bats were netted at swarming sites and/or taken by hand from the wall of summer and winter shelters. Each bat was sexed and its age estimated based on epiphyseal ossification of the thoracic limb fingers and teeth abrasion [24]. Females captured at summer colonies were checked for signs of lactation (milk production) and suckling by young animals. The forearm length was measured with callipers and the body mass determined using a portable top-loading balance. The body mass index (BMI) was calculated as body mass in grams divided by the left forearm length in millimetres [25]. A key was used to identify bat species [26]. To exclude diseased bats from the blood profile analysis, all bats sampled at hibernacula were examined for white-nose syndrome skin lesions using a UV lamp. Those showing less than 300 skin lesions on both wings were only included in the study to report reference ranges [15]. Bats used for blood sample collection and analysis were not re-captured.

### Blood analysis

To obtain the blood samples, we first disinfected the skin over the uropatagial vessel and then collected a 100 μl blood sample with a heparinised pipette tip [15]. Each blood sample was taken within 60 minutes of bat capture. Using an i-STAT portable clinical analyser (EC8+ diagnostic cartridge, Abaxis, Union City, CA, USA) we measured haematocrit (Hct, L/L), haemoglobin (Hb, g/L), sodium (Na, mmol/L), potassium (K, mmol/L), chloride (Cl, mmol/L), blood urea nitrogen (BUN, mmol/L), glucose (GLU, mmol/L), pH, partial dissolved carbon dioxide (pCO_2_, kPa), total dissolved carbon dioxide (tCO_2_, mmol/L), bicarbonate (HCO_3_, mmol/L), base excess (BE, mmol/L) and anion gap (AnGap, mmol/L). Where potassium and chloride measurements read as values higher than 9 and 140, respectively, the two values were substituted with 9.1 and 141. Five parameter subgroups were defined for statistical analysis, based on their medical relevance: i) blood electrolytes (sodium, potassium and chloride, anion gap, bicarbonate and pH); ii) red blood cell count and hydration (haematocrit, haemoglobin and blood urea nitrogen); iii) liver function and metabolism (blood urea nitrogen, glucose, potassium and pH); iv) kidney function (blood urea nitrogen, sodium, potassium and pH), and v) acid-base balance (bicarbonate, base excess, anion gap, partial dissolved carbon dioxide, total dissolved carbon dioxide and pH). The presence of particular parameters in the subgroups is non-exclusive as they can each mirror different aspects of animal physiology.

### Statistical analysis

Reference ranges for haematological and biochemical measurements were calculated from 98 samples, excluding two animals with missing or extreme values for some haematological parameters. As both forearm length and blood parameters in 18 specimens determined as juvenile (during swarming) or subadult (during hibernation) did not differ significantly from those of adults (t-test and ANOVA), the data were pooled for subsequent analysis. The t-test and ANOVA were also used for comparing female and male body size (forearm length, weight and BMI). We used principal component analysis (PCA) to evaluate inter-individual differences in distribution along axes linked with different parameter subgroups and to differentiate between healthy specimens and those showing blood profile disruption. Animals placed out of the 95% confidence limit (1.96 standard deviations) were excluded from subsequent analyses.

Normal distribution of variables in each dataset was re-tested using the Shapiro-Wilk test. All parameters were normally distributed with the exception of potassium (W = 0.841, p < 0.001) in females at summer colonies, urea (W = 0.803, p = 0.01) and anion gap (W = 0.810, p = 0.01) in males at swarming sites and sodium (W = 0.828, p = 0.03) in females at swarming sites. These variables were tested using the non-parametric Kruskal Wallis and Mann-Whitney U tests. Arithmetic means and 95% confidence intervals (± 1.96 standard deviations) are presented for normally distributed variables and the median and percentiles (lower 5% and upper 95%) for those variables with non-normal distribution. Effects of sex and locality were tested using one-way ANOVA with the Tukey’s HSD post-hoc test. Analyses were performed using Statistica v. 13.2 and R Studio v. 3.4.1 packages FactoMineR [27] and factoextra [28].

## Results

**Fig 2:**
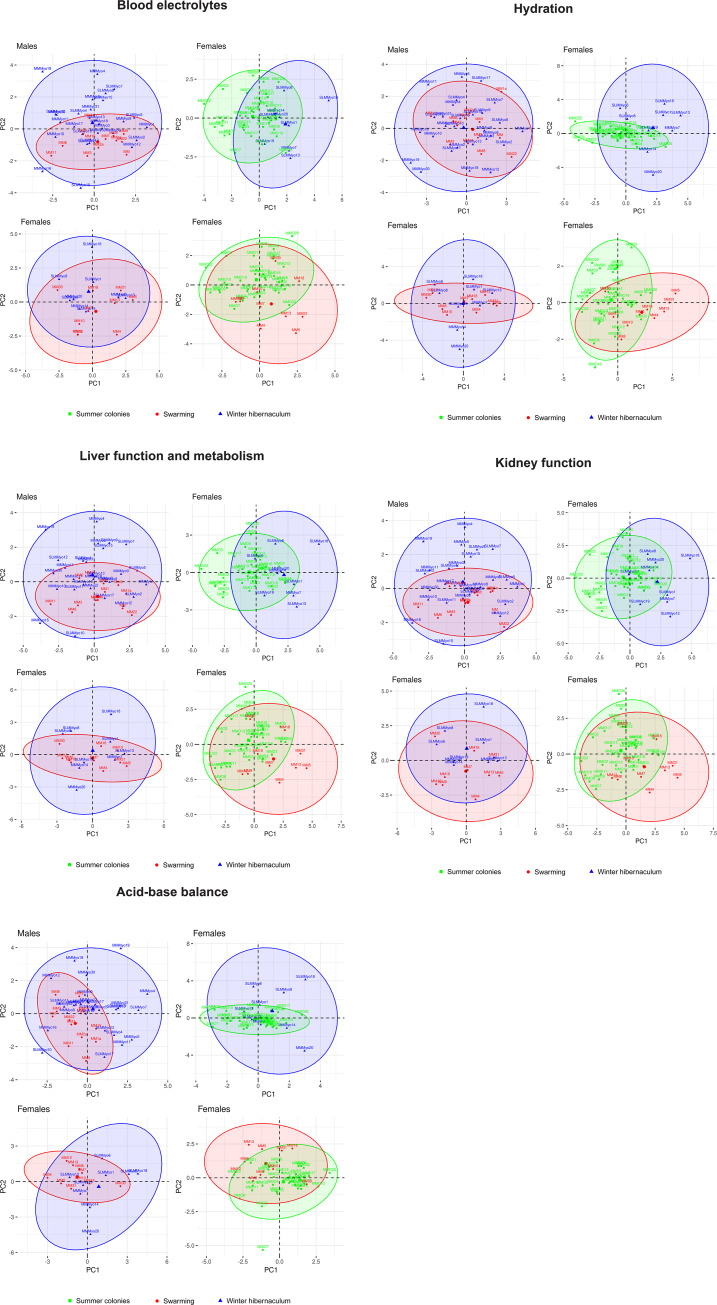
Principal component analysis for blood parameter subgroups and sampling seasons. Specimens outside the 95% confidence limits in at least one subgroup were excluded from subsequent analysis. 2A = blood electrolytes, 2B = red blood cell count and hydration, 2C = liver function and metabolism, 2D = kidney function, 2E = acid-base balance.

Greater mouse-eared bat males and females differed significantly in all three body size parameters (forearm length, weight and BMI), with larger and heavier females demonstrating sexual dimorphism (Table 1). Consequently, all subsequent analyses were undertaken separately for each sex. Principal component analyses were calculated for all combinations of blood parameter subgroups and sampling seasons and, in every case, the first two PCA components explained more than 50% of variation (Fig 2A–2E). On the other hand, there was no clear pattern in the distribution of specimens in multivariate space, i.e. it was impossible to differentiate particular groups of animals showing different physiological states in the multi-factorial space. Ten specimens were excluded as they occurred outside the 95% confidence limits in at least one parameter subgroup. Severity of blood parameter disruption in these specimens varied from signs of dehydration to potential multi-systemic disorders.

**Table 1:** Comparison of body size parameters in male and female greater mouse-eared bats (*Myotis myotis*).

Variable	Mean		t-test			Analysis of variance	
	Males	Females	t-value	df	p	F 1.94	p
Antebrachium length (mm)	59.85	62.92	-10.82	94	< 0.001	117.17	< 0.001
Body weight (g)	24.18	28.93	-7.02	94	< 0.001	49.23	< 0.001
Body mass index	0.40	0.46	-5.37	94	< 0.001	28.85	< 0.001

**Fig 3:**
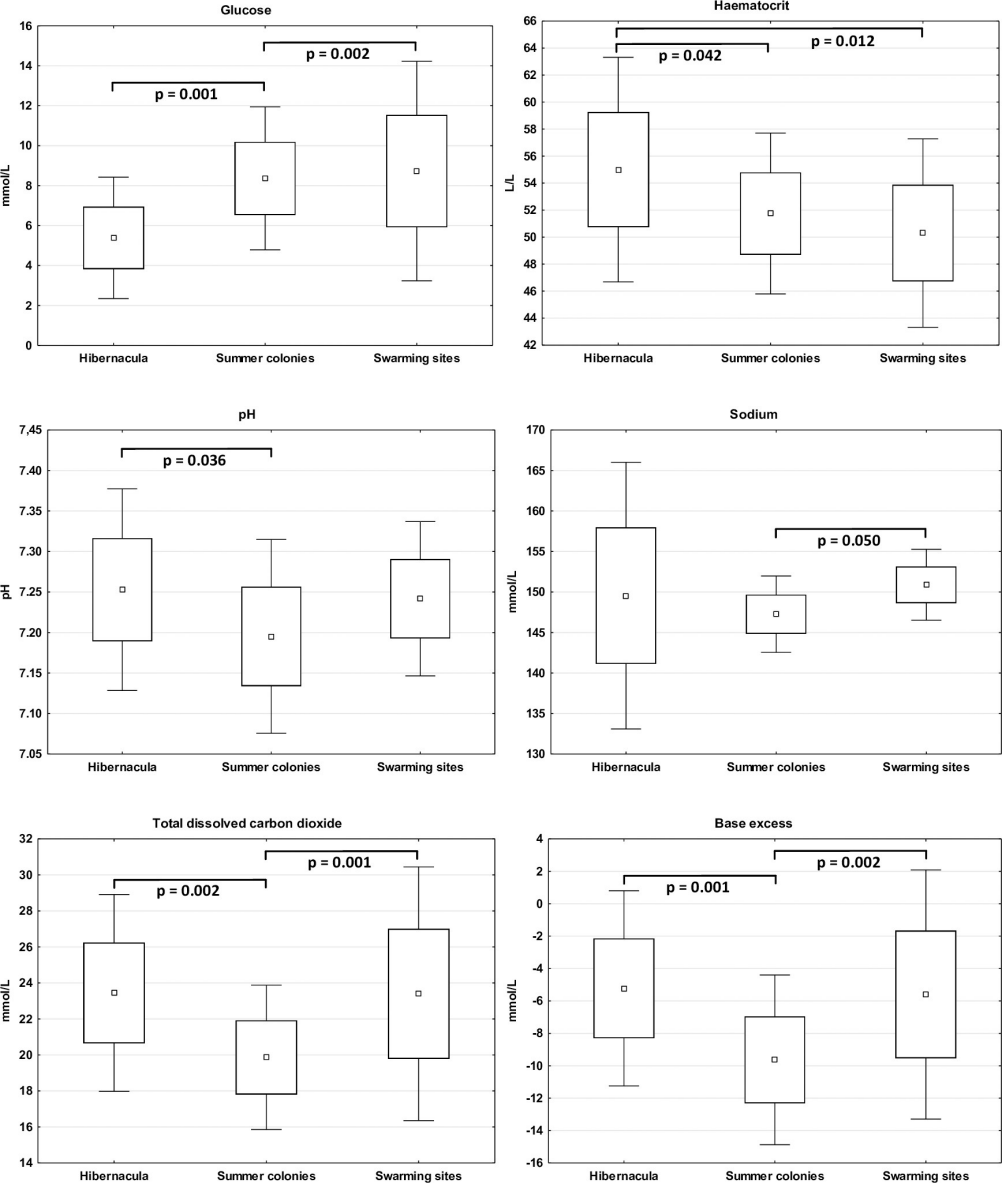
Physiological state-dependent seasonal variability in female greater mouse-eared bat (*Myotis myotis*) blood parameters. Square = mean, box = ±SD, whiskers = ±1.96*SD.

With exception of chloride, blood urea nitrogen, partial dissolved carbon dioxide and anion gap, female blood parameters differed with physiological state (Table 2). Post-hoc tests confirmed that female blood parameters were mainly impacted by physiological extremes during hibernation and lactation. Just two electrolytes reached extreme values during the swarming period, with potassium at its lowest and sodium highest. Hibernation resulted in decreased glucose levels but increased haematocrit, haemoglobin and pH. Females displayed lowest sodium, total dissolved carbon dioxide, bicarbonate and base excess levels during lactation (Fig 3). Potassium, haematocrit and haemoglobin were all influenced by male physiological state, and these showed the same trend as in females. Only haematocrit (F = 4.72, p = 0.042) and haemoglobin (F = 4.90, p = 0.039) differed between males and females during the swarming period; hence, reference values were calculated from the pooled dataset. Pooled dataset values lying outside the 95% confidence intervals were excluded from further analysis and normal haematological and biochemistry reference ranges are reported as the recalculated mean ± 95% confidence intervals for each variable and physiological state (Table 3).

**Table 2:** Greater mouse-eared bat (*Myotis myotis*) blood parameters at different physiological points in their annual life cycle.

**Variable**	**Swarming**											
	**Females**						**Males**					
	***n***	**Mean**	**Min**	**Max**	**CI**		***n***	**Mean**	**Min**	**Max**	**CI**	
Hct (L/L)	10	50.30	46.00	56.00	43.32	57.28	12	53.08	50.00	57.00	48.32	57.84
Hb (g/L)	10	170.90	156.00	190.00	147.26	194.54	12	180.50	170.00	194.00	164.39	196.61
Na* (mmol/L)	10	150.00	149.00	156,00	149.00	156.00	12	151.50	147.00	159.00	144.95	158.05
K (mmol/L)	10	5.91	3.80	9.10	2.49	9.33	12	5.93	4.40	7.90	3.83	8.04
Cl (mmol/L)	10	119.70	112.00	126.00	110.41	128.99	12	120.92	115.00	125.00	114.15	127.68
Urea* (mmol/L)	10	18.74	11.10	25.20	8.39	29.09	12	19.30	12.00	43.60	12.00	43.60
Glu (mmol/L)	10	8.73	5.90	14.70	3.23	14.23	12	7.34	2.90	12.70	1.74	12.95
pH	10	7.24	7.16	7.30	7.15	7.34	12	7.25	7.19	7.30	7.19	7.31
pCO_2_ (kPa)	10	6.75	5.88	7.70	5.62	7.87	12	6.45	5.16	8.22	5.00	7.91
tCO_2_ (mmol/L)	10	23.40	19.00	29.00	16.35	30.45	12	22.92	18.00	27.00	18.01	27.82
HCO_3_ (mmol/L)	10	21.90	17.70	26.90	15.39	28.41	12	21.38	16.40	25.40	16.68	26.08
BE (mmol/L)	10	-5.60	-11.00	0.00	-13.29	2.09	12	-5.83	-11.00	-1.00	-11.04	-0.63
AnGap* (mmol/L)	9	15.22	13.00	17.00	12.16	18.29	12	14.00	13.00	22.00	13.00	22.00
**Variable**	**Hibernation**											
	**Females**						**Males**					
	***n***	**Mean**	**Min**	**Max**	**CI**		***n***	**Mean**	**Min**	**Max**	**CI**	
Hct (L/L)	9	55.00	50.00	63.00	46.68	63.32	25	56.96	51.00	65.00	50.78	63.14
Hb (g/L)	9	187.00	170.00	214.00	158.58	215.42	25	193.68	173.00	221.00	172.64	214.72
Na (mmol/L)	9	149.56	133.00	160.00	133.10	166.02	25	151.48	138.00	161.00	139.69	163.27
K (mmol/L)	9	7.29	4.90	8.50	4.87	9.71	25	6.99	4.30	9.10	4.56	9.42
Cl (mmol/L)	9	120.67	103.00	130.00	104.39	136.95	25	123.12	109.00	136.00	108.85	137.39
Urea (mmol/L)	9	19.38	11.50	35.00	4.68	34.07	25	23.33	10.00	40.50	8.52	38.14
Glu (mmol/L)	9	5.39	3.80	8.80	2.35	8.43	25	5.74	2.70	9.80	1.79	9.69
pH	9	7.25	7.18	7.38	7.13	7.38	25	7.25	7.08	7.37	7.12	7.38
pCO_2_ (kPa)	9	6.64	4.49	8.36	4.40	8.89	25	6.43	5.20	8.91	4.77	8.10
tCO_2_ (mmol/L)	9	23.44	20.00	27.00	17.98	28.91	25	22.76	17.00	29.00	16.82	28.70
HCO_3_ (mmol/L)	9	21.83	18.10	25.40	16.39	27.27	25	21.34	16.00	28.00	15.44	27.24
BE (mmol/L)	9	-5.22	-10.00	-1.00	-11.25	0.80	25	-5.92	-13.00	3.00	-13.27	1.43
AnGap (mmol/L)	9	14.22	10.00	18.00	8.53	19.92	22	14.45	9.00	18.00	9.30	19.61
**Variable**	**Lactation**											
	**Females**						**Males**					
	***n***	**Mean**	**Min**	**Max**	**CI**		***n***	**Mean**	**Min**	**Max**	**CI**	
Hct (L/L)	28	51.75	46.00	57.00	45.79	57.71						
Hb (g/L)	28	175.89	156.00	194.00	155.81	195.98						
Na (mmol/L)	30	147.27	142.00	154.00	142.55	151.98						
K* (mmol/L)	30	8.55	5.40	9.10	5.90	9.10						
Cl (mmol/L)	28	120.82	112.00	131.00	113.04	128.61						
Urea (mmol/L)	28	24.80	6.50	45.30	3.55	46.04			**NA**			
Glu (mmol/L)	28	8.36	4.50	12.50	4.79	11.94						
pH	30	7.20	7.05	7.33	7.08	7.31						
pCO_2_ (kPa)	30	6.39	4.49	7.79	4.79	8.00						
tCO_2_ (mmol/L)	30	19.87	17.00	24.00	15.86	23.88						
HCO_3_ (mmol/L)	30	18.49	15.30	23.00	14.42	22.56						
BE (mmol/L)	30	-9.63	-15.00	-3.00	-14.87	-4.40						
AnGap (mmol/L)	19	16.53	12.00	22.00	10.49	22.56						

Hct = haematocrit, Hb = haemoglobin, Na = sodium, K = potassium, Cl = chloride, Urea = blood urea nitrogen, Glu = glucose, pH = potential of hydrogen, pCO_2_ = partial dissolved carbon dioxide, tCO_2_ = total dissolved carbon dioxide, HCO_3_ = bicarbonate, BE = base excess and AnGap = anion gap, * = non-normal distribution of parameter, CI = confidence interval, NA = non-applicable.

**Table 3:** Normal haematology and biochemistry reference ranges for the greater mouse-eared bat (*Myotis myotis*) at seasonally specific physiological states.

Variable	Swarming		Hibernation		Lactation (only females)	
	*n*	Reference values	*n*	Reference values	*n*	Reference values
Hct (L/L)	22	45.46–58.18	33	49.86–62.50	28	45.79–57.71
Hb (g/L)	22	154.52–197.75	33	169.47–212.59	28	155.81–195.98
Na (mmol/L)	21	146.33–155.38	33	139.90–163.13	29	142.96–151.11
K (mmol/L)	21	3.39–8.15	33	4.91–9.39	29	6.61–10.01
Cl (mmol/L)	22	112.46–128.26	33	109.76–136.36	26	114.64–126.90
Urea (mmol/L)	20	5.86–35.87	33	7.95–35.51	28	3.55–46.04
Glu (mmol/L)	21	2.78–12.53	32	2.17–8.63	26	5.37–11.34
pH	20	7.20–7.31	31	7.16–7.36	26	7.12–7.27
pCO_2_ (kPa)	20	5.59–7.56	31	5.05–7.78	29	4.99–7.92
tCO_2_ (mmol/L)	22	17.29–28.98	34	17.17–28.71	30	15.86–23.88
HCO_3_ (mmol/L)	22	16.14–27.10	33	15.94–26.60	28	14.75–21.59
BE (mmol/L)	22	-12.02–0.56	33	-12.37–0.37	29	-14.57 - -5.16
AnGap (mmol/L)	20	11.33–18.57	30	9.64–19.50	19	10.49–22.56
Shaded values differ statistically for males and females						
**Swarming**		**Males**		**Females**		
Hct (L/L)	12	48.32–57.84	10	43.32–57.28		
Hb (g/L)	12	164.39–196.61	10	147.26–194.54		

Hct = haematocrit, Hb = haemoglobin, Na = sodium, K = potassium, Cl = chloride, Urea = blood urea nitrogen, Glu = glucose, pH = potential of hydrogen, pCO_2_ = partial dissolved carbon dioxide, tCO_2_ = total dissolved carbon dioxide, HCO_3_ = bicarbonate, BE = base excess and AnGap = anion gap. Reference ranges are reported as the recalculated mean ± 95% confidence intervals.

## Discussion

We performed analysis of blood parameters characteristic for the greater mouse-eared bat during its annual life cycle. Despite sexual dimorphism and differences revealed by univariate analysis, the overall multivariate pattern suggests low seasonal variation. Our results indicate that homeostatic mechanisms regulate the bat’s internal conditions within narrow limits that sustain bodily functions necessary for life, irrespective of the changing bat’s physiological/ transcriptomic states [29–31].

Organismal homeostasis evolved to maintain stability for physiological functions, influencing successful performance of organisms in their environments [32]. Importantly, bats are an old evolutionary group of mammals [33] with enhanced capacity of metabolism regulation necessary for powered flight [34]. High seasonal variation in physiological factors could be damaging. Considering blood parameters measured in this study, they are essential for supporting vital functions of bats. Therefore, multiple homeostatic mechanisms including negative feedback evolved to control key physiological factors such as fluid balance, electrolyte concentrations, acid-base balance and glucose levels [32]. While some red and white blood cells associated hematological parameters (erythrocyte count, haemoglobin concentration, leukocyte total and differential counts) exerted seasonal changes in *Rhinolophus ferrumequinum*, *Miniopterus schreibersii* and *Myotis dasycneme* [35,36], similar to our findings, there were no differences in hematocrit during cold and warm seasons [35].

Regarding the high WNS prevalence and infection intensity in *Myotis myotis* bats, we may hypothesize that efficacy of blood homeostatic mechanisms allows for infection survival and enabled evolution of tolerance to *P*. *destructans* in hyperendemic Palearctic regions [15,21]. Species body size is another factor that determines differential outcomes of *P*. *destructans* infection in bat species [37]. We considered allometry [38] of blood parameters for male and female bats. Body mass analysis with respect to physiological parameters, however, proved successful only in fasting hibernating bats under pathogen pressure [15].

A sample size of least 80 to 120 observations is recommended for establishing reference values, preferably analysed using non-parametric tests and reported as 95% confidence intervals [39]. These conditions were fulfilled in this study. In addition, however, we also undertook a further critical step, i.e. definition of health. Though the bats showed no signs of clinical disease at the time of blood sampling, this was only a vague definition as we have limited data on symptoms associated with bat diseases [40]. Moreover, it was impossible to obtain an adequate sample size for males during summer as male bats remain solitary at this time. During their active period in autumn, both males and females could only be captured and sampled in sufficient numbers at swarming sites. To address spatial variation of blood parameters [41] for establishment of reference values for healthy *Myotis myotis* individuals, we sampled at six Central European localities. *Myotis myotis* bat is a regionally migrating species with regular movements associated with its annual life-cycle of about 150 km. The obtained results thus probably reflect greater spatial variation [42].

Physical restraint during sample collection from wild bats can alter some haematological and serum biochemistry parameters [43]; hence, the sooner blood is collected after capture, the less significant any alterations will be [44]. Keeping this in mind, we made every effort to reduce the duration of the procedure and stress levels while handling the bats, including the use of an on-site analyser to measure blood samples immediately upon collection.

As with all vespertilionid bats, the blood profile of the greater mouse-eared bat reflects adaptations for flight and a protein-rich diet [11], with all dietary requirements being derived from drinking water and ingestion of insect prey captured during their euthermic life cycle [45]. Insectivorous bat species in temperate regions, however, are also adapted for long periods of fasting during hibernation [46]. Protein digestion is associated with high blood urea levels synthesised in the bat’s liver, with renal clearance mechanisms, employing glomerular hyperfiltration, being used to remove the urea and control renal water loss [47]. In general, insectivorous bats adapted to a protein-rich diet maintain stable blood glucose levels, with larger species displaying lower blood glucose levels [48]. While the greater mouse-eared bat is a large species by European standards, blood glucose ranges starting close to hypoglycaemia in swarming and hibernating bats suggest that these physiological states incur high metabolic costs and/or utilisation of fuel switching [12].

High haematocrit and haemoglobin contents suggest the blood of this species has an extraordinary oxygen-carrying capacity [49–51]. Haematocrit and blood urea nitrogen values, in combination, represent a measure of the animal’s hydration state, with minerals and electrolytes being critical for maintaining the fluid and acid-base balance. Electrolyte concentrations are normally regulated within narrow ranges; however, this was not the case in greater mouse-eared bats, though the results need to be interpreted in terms of absent clinical signs and blood pH and acid-base parameters. Blood pH is influenced by the ratio of HCO_3_ and CO_2_, which depends on kidney metabolic and respiratory functioning and bodily buffering capacity. High haemoglobin levels, reflected by the base excess parameter, contribute significantly toward overall buffering capacity, while the anion gap represents the remaining unmeasured blood cations and anions, calculated from the levels of sodium, potassium, chloride and bicarbonate. In females, gestation and lactation can both result in an inadequate intake of minerals [45]. In handicapped captive noctule bats (*Nyctalus noctula*), for example, blood profile changes were clearly documented in lactating females [13]. The lack of similar changes in the blood profile of free-ranging lactating greater mouse-eared bats suggests that the physiologic responses of noctule females may be species-specific or due to inadequate nutrition under captivity. In general, therefore, our data suggest that while differences between male and female blood parameters may be minimal, they may vary significantly between vespertilionid bat species [36,52].

By allowing assessments of specific disease indicators and the bat’s general state of health and condition, reference blood values enable veterinarians in rescue centres to provide the best possible medical care [23, 40]. Vespertilionid bats in need of urgent intensive treatment frequently suffer from dehydration, exertion and traumatic injuries that result in an inability to forage and subsequent emaciation [40]. Therapy of small-sized wildlife patients is always a challenge, even for experienced veterinarians. While a prognosis can be made on the basis of blood values measured *in situ*, knowledge of ‘normal’ blood parameters could be used as a research tool for evaluating population health and as an indicator of poor nutritional status or environmental changes (e.g. habitat quality) in species of conservation concern.

As almost 100% of greater mouse-eared bats in European hibernacula are infected with the white-nose syndrome fungus [21], we decided to analyse blood samples from hibernating bats first, which would allow us to exclude any data showing serious alterations from the skin disease from the reference values [15]. In line with our previous findings, only those bats with a *P*. *destructans* infection intensity of less than 300 skin lesions on both wings were included in the present study because WNS diseased greater mouse-eared bats with higher infection intensity manifested significantly decreased body surface temperature, body mass index, glucose, pH, bicarbonate, base excess, anion gap and total dissolved carbon dioxide. On the other hand, eosinophil counts increased in *P*. *destructans* infected bats [15]. Likewise, hibernating North American little brown bats (*Myotis lucifugus*) exhibited electrolyte depletion, hypotonic dehydration and metabolic acidosis increasing with severity of wing damage by *P*. *destructans* infection [53,54]. Skin lesions on white-nose syndrome affected bats transitioning into the active period usually heal in about two to three weeks, depending on the pathological grade [19]. Nevertheless, white-nose syndrome may induce physiological carry-over effects in surviving bats [55] due to chronic stress, lowered flight performance due to wing membrane scarring and decreased foraging efficiency; euthermic bats may also be in poor health [56–61]. However, the impact of such adverse patho-physiological effects on the blood reference ranges of euthermic European greater mouse-eared bats remains to be explored.

To conclude, baseline data for the blood physiology and haematology of bats are required in order to fully understand their adaptations to extreme environmental conditions and responses to pathogenic stressors of conservation and/or zoonotic importance.

## Supporting information

S1 Table Data on sampling site, month, sex and measured blood parameters of greater mouse-eared bats used in the present study. (PDF)
