# Supplementary material for: Low seasonal variation in greater mouse-eared bat (Myotis myotis) blood parameters
Source: PLoS One. 2020 Jul 7;15(7):e0234784. doi: 10.1371/journal.pone.0234784 (PMC7340307; doi:10.1371/journal.pone.0234784)
Supplement: S1 Table — (PDF) [file pone.0234784.s001.pdf]

Locality	Month	Sex	Na	K	Cl	tCO2	urea	glu	hct	pH	pCO2	HCO3	BE	AnGAP	Hb
Hibernaculum	April	m	151	5,6	115	29	16	4,2	57	7,371	6,44	28	3	13	194
Hibernaculum	April	m	149	6	118	22	20,5	6,8	55	7,187	7,07	20,1	-8	17	187
Hibernaculum	April	m	152	6,5	120	24	20,1	7,2	56	7,346	5,64	23,2	-3	15	190
Hibernaculum	April	m	152	9,1	129	22	25,2	3,1	65	7,225	6,59	20,5	-7		221
Hibernaculum	April	m	161	6,3	135	22	27	2,7	60	7,232	6,61	20,9	-7	11	204
Hibernaculum	April	f	148	8,1	117	26	11,9	5,4	51	7,268	7,2	24,6	-2	14	173
Hibernaculum	April	m	151	4,3	119	24	15,2	5,4	56	7,262	6,64	22,5	-5	13	190
Hibernaculum	April	m	149	6,7	118	25	16,6	5,7	58	7,281	6,69	23,6	-3	14	197
Hibernaculum	April	m	149	7,7	124	18	24,9	7	53	7,244	5,2	16,9	-10	16	180
Hibernaculum	April	m	157	7,7	136	18	35,7	3,4	55	7,12	6,81	16,6	-13	12	187
Hibernaculum	April	m	143	7,1	109	26	10	7,2	54	7,244	7,48	24,2	-3	17	184
Hibernaculum	April	m	152	6,5	119	23	21,7	6,9	57	7,291	5,98	21,6	-5	18	194
Hibernaculum	April	f	143	8,2	115	22	15,7	8,8	56	7,289	5,67	20,4	-6	16	190
Hibernaculum	April	m	141	6,4	119	17	17,7	12	47	7,222	5,09	15,7	-12	13	160
Hibernaculum	April	m	146	8,5	122	21	24,6	5,2	55	7,256	5,77	19,3	-8	12	187
Hibernaculum	April	m	138	9,1	113	23	19,9	9,8	57	7,301	5,91	21,9	-5		194
Hibernaculum	April	m	134	9,1	114	16	48,9	5,8	64	7,223	4,82	14,9	-13		218
Hibernaculum	April	f	133	8,5	103	21	17,6	6,5	57	7,383	4,49	20,1	-5	18	194
Hibernaculum	April	m	142	7,7	115	17	31,2	9,4	57	7,137	6,29	16	-13	18	194
Hibernaculum	April	m	145	9,1	130	22	31,7	4,3	58	7,079	8,91	19,8	-10		197
Hibernaculum	April	f	151	7,4	124	25	26,4	4,7	54	7,225	7,48	23,2	-4	11	184
Hibernaculum	April	m	152	5,7	115	27	13,2	5,8	55	7,262	7,5	25,4	-2	17	187
Hibernaculum	April	m	159	6,3	133	21	32,5	6,6	57	7,256	5,98	19,9	-7	13	194
Hibernaculum	April	m	157	6,5	125	27	24,1	8,2	58	7,237	7,95	25,4	-2	13	197
Hibernaculum	April	f	159	5,6	130	20	18,9	4,4	54	7,179	6,49	18,1	-10	16	184
Hibernaculum	April	m	158	8,5	127	26	29,8	3,4	62	7,332	6,12	24,3	-2	15	211
Hibernaculum	April	f	155	8	127	21	35	6	59	7,181	6,86	19,3	-9	17	201
Hibernaculum	April	m	156	6,3	125	24	21,4	4,2	62	7,299	6,06	22,3	-4	15	211
Hibernaculum	April	m	156	5,8	126	21	16,9	7,3	45	7,225	6,4	19,9	-8	15	153
Hibernaculum	April	m	150	6	119	21	11,8	7,8	54	7,245	5,93	19,3	-8	17	184
Hibernaculum	April	m	159	5,7	131	18	27,5	7,1	57	7,235	5,2	16,5	-11	17	194
Hibernaculum	April	f	151	4,9	120	27	11,5	4,8	50	7,276	7,29	25,4	-1	10	170

Hibernaculum	April	m	157	6,8	126	23	20,9	5,2	61	7,278	6,22	21,9	-5	16	207
Hibernaculum	April	m	145	7,8	123	22	21,2	4,2	54	7,295	5,68	20,7	-6	10	184
Hibernaculum	April	m	157	7,3	132	24	40,5	2,7	51	7,296	6,19	22,7	-4	9	173
Hibernaculum	April	f	160	7,7	128	27	21,9	4,1	63	7,211	8,36	25,2	-3	15	214
Hibernaculum	April	f	146	7,2	122	22	15,5	3,8	51	7,264	5,96	20,2	-7	11	173
Summer colony	July	f	149	5,4	116	21	34,7	8,2	48	7,217	6,42	19,6	-8	19	163
Summer colony	July	f	148	7,2	119	23	31,3	9,3	51	7,269	6,22	21,4	-6	15	173
Summer colony	July	f	150	8,2	116	21	44,3	7,4	49	7,265	5,87	20	-7	22	167
Summer colony	July	f	146	8,5	114	23	27,2	4,5	53	7,163	7,79	21	-8	20	180
Summer colony	July	f	150	8,6	121	18	22,6	8,2	56	7,065	7,53	16,2	-14	22	190
Summer colony	July	f	150	5,9	120	19	28,6	6,2	53	7,204	6,12	18,1	-10	18	180
Summer colony	July	f	146	9,1	122	17	35,5	9,1	55	7,048	7,57	15,6	-15		187
Summer colony	July	f	150	7,3	122	18	24,1	8,2	51	7,269	4,9	16,8	-10	19	173
Summer colony	July	f	149	8	124	17	40,6	7	47	7,177	5,75	16	-12	16	160
Summer colony	July	f	149	8,8		19				7,329	4,49	17,7	-8		
Summer colony	July	f	147	7	122	19	23,3	7,5	56	7,16	6,68	17,9	-11	15	190
Summer colony	July	f	144	8,3	120	20	21,2	11,5	53	7,206	6,15	18,3	-10	14	180
Summer colony	July	f	148	8	121	20	21,5	9,2	52	7,166	6,94	18,8	-10	16	177
Summer colony	July	f	147	9,1	121	21	23,2	7,6	53	7,14	7,57	19,3	-10		180
Summer colony	July	f	150	9,1	126	19	21,5	7,2	57	7,181	6,11	17,1	-11		194
Summer colony	July	f	147	9,1	120	24	27,2	9,9	53	7,255	6,88	22,9	-4		180
Summer colony	July	f	148	9,1		21				7,153	7,43	19,5	-9		
Summer colony	July	f	146	7,9	112	24	10	10,7	53	7,316	6	23	-3	19	180
Summer colony	July	f	146	6,6	120	17	25,4	11,8	48	7,158	5,74	15,3	-13	18	163
Summer colony	July	f	148	8,1	122	14	26,4	9,8	51	7,055	6,15	12,9	-17	20	173
Summer colony	July	f	146	9,1	117	20	38,3	7,8	49	7,151	7,08	18,5	-10		167
Summer colony	July	f	154	9,1	131	17	45,3	7,3	46	7,202	5,23	15,4	-13		156
Summer colony	July	f	139	8,3	141	19	19,4	10,5	45	7,173	6,48	17,9	-11		153
Summer colony	July	f	144	7,6	116	20	10,9	10,5	53	7,155	7,12	18,8	-10	16	180
Summer colony	July	f	146	8,6	125	17	41,1	12,5	47	7,216	5,24	15,9	-12	13	160
Summer colony	July	f	150	6,8	131	15	15,2	9,2	48	7,239	4,51	14,5	-13	11	163
Summer colony	July	f	147	8,9	124	20	14,8	6,6	49	7,216	6,21	18,9	-9	13	167
Summer colony	July	f	144	9,1	124	20	11,6	7	56	7,184	6,46	18,2	-10		190

Summer colony	July	f	146	7,7	124	18	17,8	7,2	53	7,163	6,09	16,4	-12	14	180
Summer colony	July	f	146	9,1	123	19	16,2	8,3	49	7,201	6,16	18,1	-10		167
Summer colony	July	f	147	8,6	123	22	16	7,9	52	7,217	6,85	20,9	-7	12	177
Summer colony	July	f	144	9,1	122	16	16,4	9,2	54	7,121	6,13	15	-14		184
Summer colony	July	f	142	8,3	118	21	6,5	9	54	7,224	6,31	19,5	-8	13	184
Summer colony	July	f	147	8,9	122	17	14,2	5,5	59	7,04	7,57	15,4	-15	19	201
Summer colony	July	f	146	9,1	122	21	13,6	6,6	53	7,188	6,89	19,6	-9		180
Summer colony	July	f	150		124	13	28,3	7,4	46	6,94	6,82	11	-21		156
Swarming site	September	m	156	6,9	128	26	23,6	2,9	53	7,317	6,45	24,8	-1	10	180
Swarming site	September	m	155	6,7	125	23	20,1	3,7	52	7,245	6,66	21,6	-6	15	177
Swarming site	September	f	150	5,3	117	25	12	9,4	46	7,279	6,58	23,1	-4	16	156
Swarming site	September	f	151	4,5	112	29	12,5	6,1	49	7,294	7,4	26,9	0	17	167
Swarming site	September	m	159	5,6	123	21	43,2	10,6	50	7,252	5,91	19,5	-8	22	170
Swarming site	September	f	149	6,5	121	23	25,2	5,9	49	7,242	6,6	21,3	-6	13	167
Swarming site	September	f	152	7,8	126	20	22,1	8,1	48	7,246	5,88	19,1	-8	15	163
Swarming site	September	m	149	7,5	119	25	15,7	6,8	56	7,188	8,22	23,5	-5	14	190
Swarming site	September	f	150	7,5	121	21	18	7,9	51	7,25	5,94	19,5	-8	17	173
Swarming site	September	m	152	5,7	125	18	18,8	12,7	50	7,233	5,16	16,4	-11	16	170
Swarming site	September	f	153	5,2	124	19	21,6	6,7	46	7,159	6,65	17,7	-11	16	156
Swarming site	September	f	156	4,2	119	27	22,3	8,3	50	7,248	7,7	25,2	-2	16	170
Swarming site	September	m	152	4,9	119	22	24,2	6,2	57	7,264	6,09	20,7	-6	18	194
Swarming site	September	m	149	5,2	118	26	15,3	6,6	52	7,289	6,7	24,1	-2	13	177
Swarming site	September	m	150	5	120	24	17,7	2,9	54	7,261	6,59	22,2	-5	13	184
Swarming site	September	m	151	6,5	125	21	17,8	6,9	55	7,278	5,73	20,1	-7	13	187
Swarming site	September	m	147	6,4	121	21	19,8	9,3	51	7,212	6,45	19,5	-8	13	173
Swarming site	September	m	148	4,4	117	24	12	9,4	56	7,269	6,42	22,1	-5	14	190
Swarming site	September	m	152	7,9	124	23	36,4	4,8	52	7,244	6,64	21,5	-6	14	177
Swarming site	September	f	150	5,2	119	24	18,1	7,8	56	7,236	7,13	22,7	-5	13	190
Swarming site	September	m	154	5,4	115	27	43,6	8,2	52	7,3	6,87	25,4	-1	19	177
Swarming site	October	f	149	9,1	125	19	24,5	12,4	56	7,161	6,68	17,9	-11		190
Swarming site	October	f	149	3,8	113	27	11,1	14,7	52	7,302	6,9	25,6	-1	14	177
